# *Ficus deltoidea* suppresses endothelial activation, inflammation, monocytes adhesion and oxidative stress via NF-κB and eNOS pathways in stimulated human coronary artery endothelial cells

**DOI:** 10.1186/s12906-020-2844-6

**Published:** 2020-02-17

**Authors:** Amirah Mohd Ariff, Nurul Ain Abu Bakar, Suhaila Abd. Muid, Effat Omar, Nor Hadiani Ismail, Abdul Manaf Ali, Noor Alicezah Mohd Kasim, Hapizah Mohd Nawawi

**Affiliations:** 1 Institute of Pathology, Laboratory and Forensic Medicine (I-PPerForM), Universiti Teknologi MARA (UiTM), Sungai Buloh Campus, 47000 Sungai Buloh, Selangor Malaysia; 2 Faculty of Medicine, Universiti Teknologi MARA (UiTM), Sungai Buloh Campus, Sungai Buloh, Selangor Malaysia; 3 Atta-ur-Rahman Institute for Natural Product Discovery, Universiti Teknologi MARA (UiTM), Puncak Alam Campus, 42300 Bandar Puncak Alam, Selangor Malaysia; 4 Faculty of Bioresources and Food Industry, Universiti Sultan Zainal Abidin (UniSZA), 20300 Kuala Terengganu, Terengganu Malaysia

**Keywords:** Atherosclerosis, *Ficus deltoidea*, Inflammation, Endothelial activation, Monocyte adhesion, Oxidative stress

## Abstract

**Background:**

*Ficus deltoidea* (FD) has been shown to have antidiabetic, anti-inflammatory, antinociceptive and antioxidant properties. However, its effects on key events in the pathogenesis of atherosclerosis are unknown.

**Aim:**

To investigate the endothelial activation, inflammation, monocyte-endothelial cell binding and oxidative stress effects of four FD varieties.

**Methods:**

Human coronary artery endothelial cells (HCAEC) were incubated with different concentrations of aqueous ethanolic extracts of FD var. *trengganuensis* (FDT), var. *kunstleri* (FDK), var. *deltoidea* (FDD) and var. *intermedia* (FDI), together with LPS. Protein and gene expression of vascular cell adhesion molecule-1 (VCAM-1), intercellular cell adhesion molecule-1 (ICAM-1), endothelial-leukocyte adhesion molecule-1 (E-selectin), interleukin-6 (IL-6), Nuclear factor-κB (NF-κB) p50 and p65 and endothelial nitric oxide synthase (eNOS) were measured using ELISA and QuantiGene plex, respectively. Adhesion of monocyte to HCAEC and formation of reactive oxygen species (ROS) were detected by Rose Bengal staining and 2′-7′-dichlorofluorescein diacetate (DCFH-DA) assay.

**Results:**

FDK exhibited the highest inhibition of biomarkers in relation to endothelial activation and inflammation, second in reducing monocyte binding (17.3%) compared to other varieties. FDK (25.6%) was also the most potent at decreasing ROS production.

**Conclusion:**

FD has anti-atherogenic effects, possibly mediated by NF-κB and eNOS pathways; with FDK being the most potent variety. It is potentially beneficial in mitigating atherogenesis.

## Background

Atherosclerosis is an insidious disease of large and medium-sized arteries; characterised by accumulation of inflammatory cell, foamy macrophages, lipid and smooth muscle cells within the intima of the arteries [1]. This condition underlies cardiovascular diseases such as stroke and coronary artery disease; the primary cause of mortality and morbidity worldwide [2]. Further, it has a strong association with hypertension and diabetes mellitus, two diseases with increasing trend globally, leading to heightened burden on the healthcare system [2, 3]. In Malaysia, cardiovascular disease is the major cause of death, contributing to 35%, of overall mortality in 2016 [4].

The pathogenesis of atherosclerosis is substantially contributed by endothelial activation, inflammation, monocyte-endothelial cell binding and oxidative stress. Endothelial injury caused by certain conditions such as hypertension or cigarette smoking, leads to endothelial cell overexpression of pro-inflammatory cytokines (interleukin-6, IL-6) and adhesion molecules (vascular cell adhesion molecule-1 (VCAM-1); intercellular cell adhesion molecule-1 (ICAM-1); endothelial-leukocyte adhesion molecule-1 (E-selectin)). This activation has been reported to be mediated via nuclear factor-κB (NF-κB) [5, 6]. As a result, there is increased binding of circulating monocytes to endothelial cells and subsequent migration into the intima [7]; an important early event in the pathogenesis of atherosclerosis [6, 8]. Inflammation is known to suppress endothelial nitric oxide synthase (eNOS), resulting in mass production of reactive oxygen species (ROS) and thus increased oxidative stress [9]. The elevated expression of IL-6 and ICAM-1 in the above process could be used as surrogate markers of endothelial activation to reflect the processes of atherosclerosis formation and progression [10]. In addition, it could also be suggested that supplementation with an endothelial activation suppressor agent with anti-inflammatory, anti-monocyte binding and anti-oxidant properties may be able to mitigate atherogenesis.

*Ficus deltoidea* Jack (Moraceae) is a notable plant in the Malay traditional healing practice. It is an evergreen shrub, raising about two metres tall with grey bark and spoon-shaped leaves. Out of 15 recognized varieties, seven of the varieties occur in Peninsular Malaysia; namely var. *deltoidea*, var. *angustifolia*,var. *trengganuensis*, var. *bilobata*, var*. intermedia*, var. *kunstleri* and var. *motleyana* [11]. However, current study was carried out on four varieties (var. *trengganuensis*, var. *kunstleri*, var. *deltoidea* and var. *intermedia*) only because these varieties are the most abundant available in Peninsular Malaysia. Majority of the varieties grow in lowlands at below 1200 m altitude, except var. *intermedia* which can be found in the mountain regions.

In Malay traditional medicine, concoction of its leaves was used as an anti-diabetic remedy as well as boost blood circulation, regain energy and improve sexual function [12]. Chemical constituents of FD were determined to be from three major groups; phenylisopropanoid (coumaric acid, caffeic acid and ferulic acid), phenolic compounds (vitexin, isovitexin, catechins, naringin, flavones, anthocyanins and proanthocyanins) and tannins (gallic acid and ellagic acid) [13, 14]. Vitexin and isovitexin are two active compounds isolated from FD; they have been reported to significantly lower the levels of postprandial blood glucose of normoglycaemic mice and diabetic rats [15]. Both primary metabolites of FD vitexin and isovitexin have also been reported to be responsible for the anti-inflammatory effects [13]. Nevertheless, little is known about its effects on the development of atherosclerosis. Leaves of the plants are used because it is the part of the plant has been the most investigated.

This study aims to investigate effects of four FD varieties on endothelial activation, inflammation, monocytes binding to endothelial cells and oxidative stress in human endothelial cells, as well as to identify possible underlying mechanisms in mediating those effects.

## Methods

### Preparation of FD leaf extract

Leaves from FD var. *trengganuensis* (FDT), FD var. *kunstleri* (FDK) and FD var. *deltoidea* (FDD) were obtained from Kuala Terengganu, Terengganu, Malaysia under voucher specimen numbers: 00366, 00048, and 00050 respectively. FD var. *intermedia* (FDI) was collected from Cameron Highland, Pahang, Malaysia under voucher specimen number: 00303. Professor Nashriyah Mat of School of Agricultural Science and Biotechnology, Universiti Sultan Zainal Abidin (UniSZA) identified the leaves of the plant and the specimens were deposited in UniSZA’s herbarium. The aqueous ethanolic FD extracts were prepared by our team from Atta-ur-Rahman Institute of Natural Product Discovery (AuRIns), Universiti Teknologi MARA (UiTM), Puncak Alam Campus, Selangor, Malaysia. The leaves were dried, milled, weighed and soaked in 50% ethanol (50:50; ethanol:water (v/v)) for 24 h. Then, the material was sonicated for 30 min at 40 °C, filtered using vacuum filter and rotary evaporated at 40 °C. The crude extracts were then freeze-dried to a dark green paste and stored at 4 °C until use.

### Cell culture

Human coronary artery endothelial cells (HCAEC) provided by Lonza, Allendale, USA were cultured in endothelial growth medium (EGM) until confluent, at 37 °C in a humidified incubator set at 5% carbon dioxide (CO_2_). The cells were collected by Accutase and subcultured at a 1:3 (culture:medium) ratio. All experiments were performed with HCAEC from passage five. RAW 264.7 cells obtained from American Type Culture Collection (ATCC), Manassas, USA, were cultured in Dulbecco’s Modified Eagle Media, DMEM (Cellgro, Manassas, USA) at 37 °C in a humidified incubator set at 5% carbon dioxide (CO_2_). Experiment on oxidative stress was performed with RAW264.7 from passage four.

### Cytotoxicity assay

The cytotoxicity effect of FD extracts on HCAEC cells was tested using 3-(4,5-dimethylthiazol-2-yl)-2,5-diphenyl tetrazolium bromide (MTT) assay. About 1 × 10^4^ cells/well were dispensed into a 96-well culture plate and incubated with various concentrations of the four different crude extracts (3.9–500 μg/ml). The cells were incubated at 37 °C for 24 h in humidified 5% CO_2_ environment. Then, 20 μl of MTT solution (Invitrogen, Rockville, USA) (5 mg/ml phosphate-buffered saline, PBS) was added to each well and incubated at 37 °C for another 4 h. The incubation medium was removed and the insoluble purple formazan product formed was dissolved in 100 μl dimethyl sulfoxide (DMSO). MTT reduction was quantified by measuring the absorbance at 540 nm using a microplate reader (Micro Quant, Biotek Instruments, Winooski, USA). Toxicity of FD on RAW 264.7 was determined using similar method.

### Treatment of HCAEC with different FD varieties

All four FD varieties were diluted into various working concentrations (5.0–10.0 μg/ml) with culture media. The confluent cells of HCAEC were treated with different concentrations of each FD extract (5- 40 μg/ml) together with 1 μg/ml of lipopolysaccharide (LPS), and followed by 16 h incubation at 37 °C, 5% CO_2_. The treatments were given as follows:

- UNT: untreated cells; HCAEC neither stimulated with LPS nor treatment of FD.
- LPS: negative control; HCAEC stimulated with LPS but no FD extract added.
- LPS + FDT/FDK/FDD/FDI (5- 40 μg/ml): treatment groups; HCAEC stimulated with LPS and treated with FDT/FDK/FDD/FDI of different concentrations (5- 40 μg/ml).

LPS was used to imitate the releasing of inflammatory mediator that contributes to the pathological process of atherogenesis. The media from each sample was collected at the end of the incubation period and subjected to the following tests.

### Measurement of ICAM-1, VCAM-1, E-selectin and IL-6 protein

Concentrations of soluble adhesion molecules (ICAM-1, VCAM-1, E-selectin) and inflammation (IL-6) biomarkers in the culture media were measured using a standard ELISA kit (eBioscience, Affymetrix, Santa Clara, USA), according to manufacturer’s instructions. The absorbance was read at 405 nm using a spectrophotometer (Micro Quant, Biotek Instruments, Winooski, USA).

### Measurement of NF-κB (p50 and p65) protein in cell lysates

Stimulated HCAEC were harvested after the incubation and subjected to nuclear extraction process using Nuclear Extraction Kit (Cayman, Ann Arbor, USA); the extracts were then quantified by ELISA method using NF-κB p50 and p65 Transcription Factor Assay Kit (Cayman, Ann Arbor, USA). All procedures were performed based on the manufacturer’s instructions. The absorbance of the samples was read at 450 nm.

### Measurement of eNOS protein in cell lysates

The eNOS protein expression in cell lysates was quantitated using eNOS immunoassay kit (R&D BioSystems, USA). All procedures were conducted as suggested by the manufacturer. A microplate reader (Tecan Safire, Männedorf, Switzerland) set at 450 nm, with reference wavelength set at 570 nm was used to read the absorbance.

### Quantitation of inflammation, endothelial activation, NF-κB and eNOS genes

The RNA was extracted from cell pellets; QuantiGene Plex 96-well Assay was performed according to the manufacturer’s manual. In triplicate, RNA was transferred to the assay hybridisation plate, containing working bead mix and probe sets. Hybridisation was performed for 20 h at 54 °C ± 1 °C, with shaking at 600 rpm. Next, the mixtures were transferred to a 96-well magnetic separation plate. The beads were hybridised with preamplifier probe, amplifier probe, followed by label probe, and finally Streptavidin-conjugated R-Phycoerythrin (SAPE). SAPE fluorescence was measured with Luminex FlexMap three-dimensional instrument (Luminex Corp., Austin, TX) to indicate the amount of mRNA transcripts captured by the beads. The target-specific RNA molecules of LPS-stimulated HCAEC were: IL-6: NM_000600; ICAM-1: NM_000201; VCAM-1: NM_001078; E-selectin: NM_000450; NF-κB p50: NM_ 001165412.1; NF-κB p65: AF018253; eNOS: NM_000603. The geometric means of three reference genes [Glyceraldehyde-3-phosphate dehydrogenase (GAPDH): NM_002046; Hypoxanthine phosphoribosyltransferase 1 (HPRT1): NM_000194; Glucuronidase beta (GUSB): NM_000181] were used for normalisations. Fold-changes were taken as the relative ratios between the normalised reference values of all treatment groups and the values of the untreated group.

### Monocyte binding assay

Adherence of monocytic cells U937 (ATCC, Manassas, USA) to HCAEC was performed using the Rose Bengal method (Gamble & Vadas, 1988). Treated and untreated LPS-stimulated HCAEC cultures (1 × 10^4^ cells/well) grown in 96-well microplate were incubated in a medium supplemented with different concentrations of FD varieties for 16 h at 37 °C. Then U937 cells (5 × 10^5^ cells/ml) were distributed into each well, followed by 1 h incubation at 37 °C; 5% CO_2_. Then 100 μl of a 0.25% solution of Rose Bengal stain in PBS was added to each well and incubated at 25 °C for 10 min. Excess stain was removed with PBS (supplemented with 10% fetal bovine serum, FBS) before 200 μl of ethanol: PBS solution (1:1 v /v) was added and incubated further for 1 h at 25 °C. The absorbance was measured at 570 nm wavelength.

### Measurement of ROS production in stimulated RAW 264.7

The effect of varying concentrations of FD on oxidative stress was quantitated by 2′-7′-dichlorofluorescein diacetate (DCFH-DA) inhibition in RAW 264.7 cell line. RAW 264.7 murine macrophage cells were used instead of HCAEC was due to its ability to produce high amount of ROS after an oxidant challenge. It is often the common cells used when it comes to studying ROS-mediated cellular events [16], as macrophages are the major sources of oxidative stress. The cells were seeded on a 96-well cell culture plate and stimulated with 20 μg/ml LPS together with 400 U/ml recombinant mouse IFN-γ; 50 μl of four FD varieties at different concentrations (15.6, 31.3, 62.5, 125.0, 250.0 and 500 μg/ml) was added into the mix; followed by an 18-h incubation at 37 °C, 5% CO_2_. IFN- γ is the typical macrophage stimulator that exhibits a synergistic effect along with microbial products such as LPS to drive high output of ROS, nitric oxide (NO) and pro-inflammatory cytokines. At the end of the incubation, 10 μl of 10 mM DCFH-DA was added and the fluorescence intensity was recorded by a fluorescent microplate reader at 5-min intervals for 30 min at the wave of excitation (485 nm) and emission (530 nm).

### Statistical analysis

Results are reported as mean ± standard deviation (SD). They were analysed using one-way analysis of variance (ANOVA) followed by Bonferroni post-hoc analysis. Two sided *p* < 0.05 was considered significant. The percentage (%) inhibition against LPS controls for each biomarker was obtained from area under the curve (AUC) analysis for each FD extract using Graph Version 4.3.

## Results

### Cytotoxic effects of FD on HCAEC and RAW 264.7 cells

**Fig. 1:**
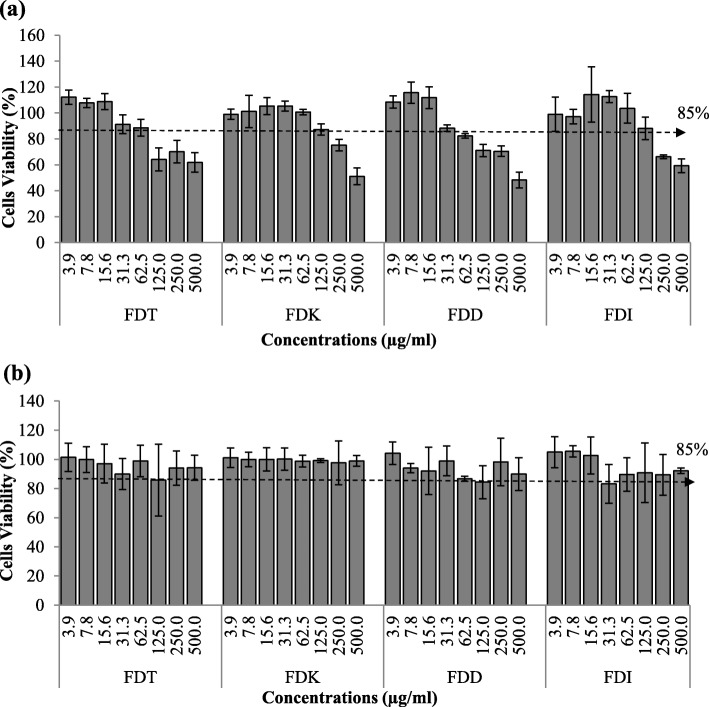
Cytotoxic effects of FDT, FDK, FDD and FDI on of HCAEC and RAW 264.7 cells. The viability of (**a**) HCAEC is good until FD concentration of 40 μg/ml; whilst (**b**) RAW 264.7 remains good at 500 μg/ml. Results are presented as percentage (%) of controls (untreated cells). Data are expressed as mean ± SD (*n* = 3). Abbreviation: FDT (FD *var. trengganuensis*), FDK (FD *var. kunstleri*), FDD (FD *var. deltoidea*), FDI (FD *var. intermedia*)

Incubation of HCAEC with all FD varieties exhibited more than 85% cell viability up to 40 μg/ml, after which the cell viability started to fall below the line (Fig. 1a). Therefore, no greater than 40 μg/ml of FD concentrations were subsequently used on HCAEC in this study. Meanwhile, RAW 264.7 cells show good cell viability (above 85%) with incubation of up to 500 μg/ml of FD, indicating low cytotoxicity of FD on RAW 264.7 cells (Fig. 1b).

### Effects of FD on adhesion molecules protein expression

**Fig. 2:**
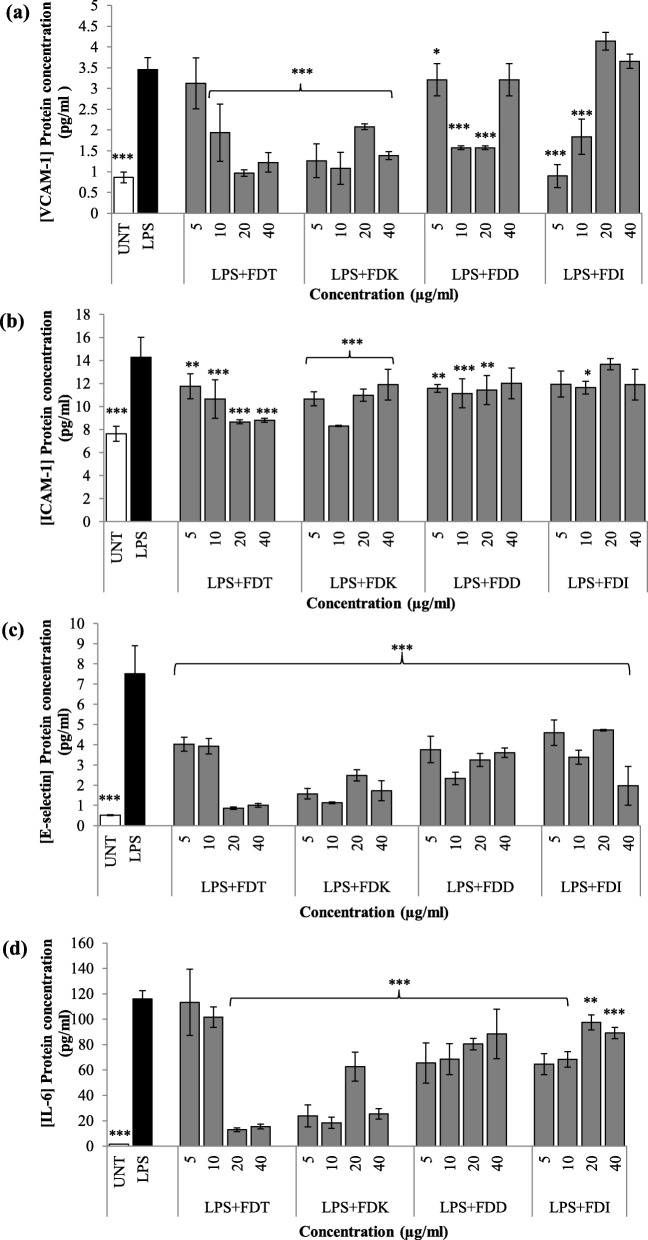
Effects of FD extracts on **a**) VCAM-1, **b**) ICAM-1, **c)** E-selectin, **d**) IL-6 protein expression in LPS-stimulated HCAEC. Before incubation, protein expression in the media was measured by ELISA. Data are expressed as mean ± SD (n = 3). Statistical analysis: ANOVA, post-hoc with Bonferroni correction; *p < 0.05, **p < 0.01 and ***p < 0.001 compared to HCAEC incubated with LPS alone. Abbreviation: UNT (untreated), LPS (lipopolysaccharide), FDT (FD var. *trengganuensis*), FDK (FD var. *kunstleri*), FDD (FD var. *deltoidea*), FDI (FD var. *intermedia*)

All four FD varieties exhibit significant reducing effects on VCAM-1, ICAM-1 and E-selectin on HCAEC cells upon co-incubation with LPS compared to LPS controls, Fig. 2a, b and c. Both FDK and FDT exhibited VCAM-1 reduction across all concentrations (*p* < 0.001), except for FDT at 5 μg/ml. FDD reduced VCAM-1 at 5–20 μg/ml (*p* < 0.05 and *p* < 0.001), whilst FDI at 5–10 μg/ml only (*p* < 0.001). According to AUC analysis, the two most potent FD varieties for the inhibition of VCAM-1 protein expression were FDT (59.4%), followed by FDK (52.8%) (Fig. 2a, Table 1).

**Table 1:** Percent of inhibition of inflammation, endothelial activation, NF-κB and eNOS biomarkers by each FD variety based on area under the curve (AUC) analysis

	VCAM-1% inhibition		ICAM-1% inhibition		E-selectin % inhibition		IL-6% inhibition		NF-κB p50% inhibition			NF-κB p65% inhibition			eNOS % increment	
	P	G	P	G	P	G	P	G	P (N)	P (C)	G	P (N)	P (C)	G	P	G
FDT	**59.4**	**77.7**	**33.8**	8.1	**75.4**	**76.5**	**66.0**	**69.2**	**6.4**	**10.3**	**85.3**	**39.4**	10.4	200.9	**15.7**	−100.1
FDK	**52.8**	**82.0**	**24.5**	**22.1**	**74.3**	**75.2**	**65.5**	**67.3**	5.1	**10.3**	**87.6**	**34.9**	**16.8**	**330.8**	**15.1**	**−100.0**
FDD	38.8	46.3	18.1	−8.2	56.3	27.3	31.5	39.1	**10.0**	**12.6**	72.1	7.6	**19.5**	**1046.1**	−4.8	**−99.1**
FDI	4.7	53.5	10.5	**10.8**	49.7	74.9	25.3	60.9	4.3	8.0	82.8	11.1	12.4	103.8	2.6	−100.1

*FDT* FD var. *trengganuensis*, *FDK* FD var. *kunstleri, FDD* FD var. *deltoidea, FDI* FD var. *intermedia*, *P* protein, *G* gene, *N* nuclear lysate, *C* cytoplasmic lysate, bold, first and second most potent FD varieties

Similarly, both FDT and FDK exhibited ICAM-1 reduction across all concentrations compared to LPS controls (*p* < 0.01 and *p* < 0.001). FDD reduced ICAM-1 at 5–20 μg/ml (*p* < 0.01 and *p* < 0.001), whilst FDI at 10 μg/ml only (*p* < 0.05). The two most potent FD var. for ICAM-1 inhibition were FDT (33.8%), followed by FDK (24.5%) (Fig. 2b, Table 1).

All four FD varieties across all doses (5–40 μg/ml) showed E-selectin reduction compared to LPS controls (*p* < 0.001). The two most potent FD varieties for E-selectin protein expression inhibition were FDT (75.4%), followed by FDK (74.3%) (Fig. 2c, Table 1).

### Effects of FD extracts on IL-6 protein secretion

All four FD varieties across all doses (5–40 μg/ml) showed IL-6 reduction compared to LPS controls (*p* < 0.01 and *p* < 0.001), except for FDT at lower concentrations (5 and 10 μg/ml). The two most potent FD var. for IL-6 protein expression inhibition were FDT (66.0%), followed by FDK (65.5%) (Fig. 2d, Table 1).

### Effects of FD on adhesion molecules gene expression

**Fig. 3:**
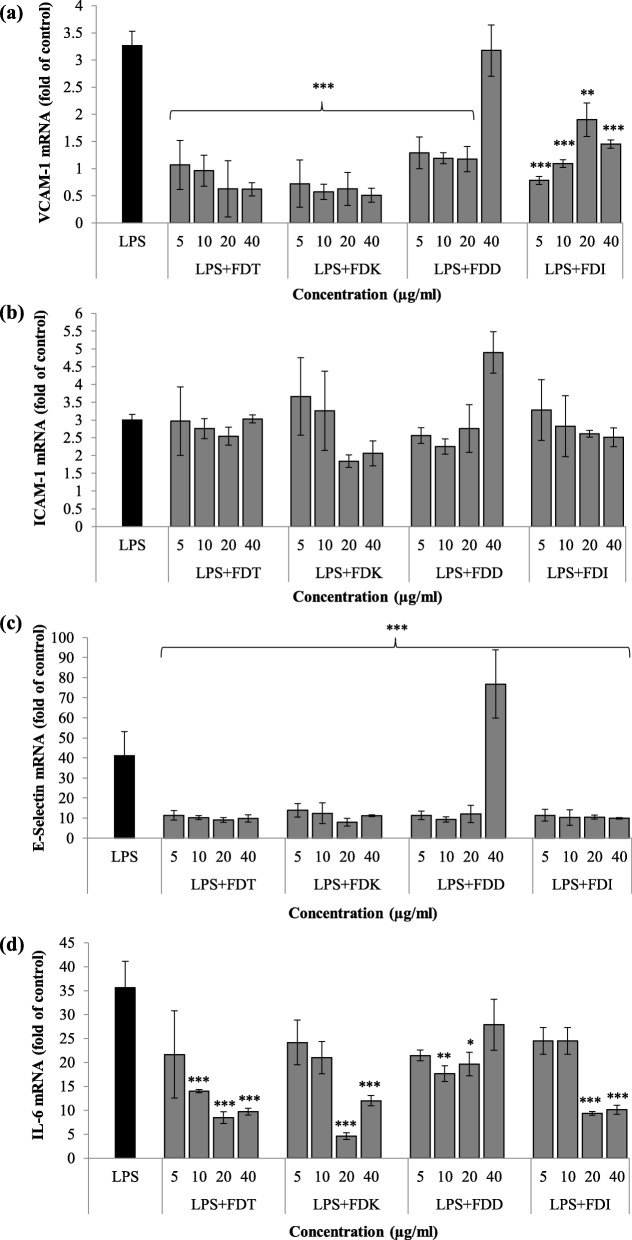
Effects of FD on **a**) VCAM-1, **b**) ICAM-1, **c**) E-selectin, **d**) IL-6 gene expression in LPS-stimulated HCAEC cell pellets. Data are expressed as mean ± SD (n = 3). Statistical analysis: ANOVA, post-hoc with Bonferroni correction; *p < 0.05, **p < 0.01 and ***p < 0.001 compared to HCAEC incubated with LPS alone. Abbreviation: LPS (lipopolysaccharide), FDT (FD var. *trengganuensis*), FDK (FD var. *kunstleri*), FDD (FD var. *deltoidea*), FDI (FD var. *intermedia*)

The co-incubation of LPS and all FD varieties down-regulated VCAM-1 mRNA expression compared to LPS controls at 5 to 40 μg/ml (*p* < 0.01 and *p* < 0.001), except FDD at the highest dose (40 μg/ml). The two most potent FD varieties for VCAM-1 gene expression down-regulation were FDK (82.0%) followed by FDT (77.7%) (Fig. 3a, Table 1).

All concentrations of all four varieties showed neutral effects on ICAM-1 mRNA level in LPS-stimulated HCAEC. However, higher concentrations of FDK and FDI suppressed the mRNA level of ICAM-1 when compared to the LPS only group, although not significant. The two most potent FD varieties for ICAM-1 gene expression down-regulation were FDK (22.1%), followed by FDI (10.8%) (Fig. 3b, Table 1).

There were significant down-regulation of E-selectin mRNA expression by the co-incubation with all FD varieties at 5 to 40 μg/ml (*p* < 0.001), except FDD at 40 μg/ml. The two most potent FD varieties for E-selectin gene expression down-regulation were FDT (76.5%), followed by FDK (75.2%) (Fig. 3c, Table 1).

### Effects of FD varieties on IL-6 gene expression

All concentrations of all four FD varieties significantly suppressed IL-6 mRNA expression (*p* < 0.05, *p* < 0.01 and *p* < 0.001) except at certain concentrations. FDT (5 μg/ml), FDK (5 and 10 μg/ml), FDD (5 and 40 μg/ml) and FDI (5 and 10 μg/ml) showed the trend of reduction of IL-6 mRNA expression when compared to LPS controls, although not significant. The two most potent FD varieties for IL-6 gene expression down-regulation were FDT (69.2%) and FDK (67.3%) (Fig. 3d, Table 1).

### Effects of FD varieties on NF-κB protein and gene expression

**Fig. 4:**
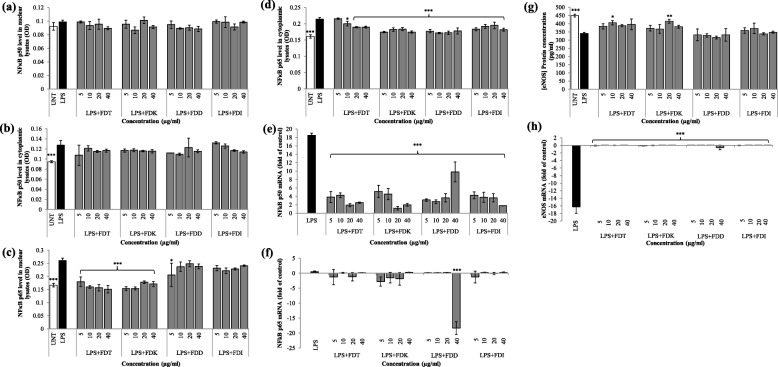
Effects of FD on **a**) NF-κB p50 in nuclear lysate protein expression, **b**) NF-κB p50 in cytoplasmic lysate protein expression, **c**) NF-κB p65 in nuclear lysate protein expression, **d**) NF-κB p65 in cytoplasmic lysate protein expression, **e**) NF-κB p50 gene expression in cell pellets, **f**) NF-κB p65 gene expression in cell pellets, **g**) eNOS protein expression in cell lysate, and **h**) eNOS gene expression in cell pellets in LPS-stimulated HCAEC. Data are expressed as mean ± SD (n = 3). Statistical analysis: ANOVA, post-hoc with Bonferroni correction; *p < 0.05, **p < 0.01 and ***p < 0.001 compared to HCAEC incubated with LPS alone. Abbreviation: UNT (untreated), LPS (lipopolysaccharide), FDT (FD var. *trengganuensis*), FDK (FD var. *kunstleri*), FDD (FD var. *deltoidea*), FDI (FD var. *intermedia*)

There were no significant reductions of NF-κB p50 activation in both nuclear and cytoplasmic lysates by all varieties across all concentrations (5–40 μg/ml) compared to LPS-stimulated HCAEC (Fig. 4a and b).

There were significant reductions in almost all concentration ranges tested in nuclear lysates with the presence of FDT and FDK (*p* < 0.001). FDD (except the lowest concentration of 5 μg/ml) and FDI showed neutral effects on the inhibition of NF-κB p65 level (Fig. 4c). In cytoplasmic lysates, the levels of NF-κB p65 were significantly reduced by almost all concentrations of all four varieties (*p* < 0.05 and *p* < 0.001), except FDT at 5 μg/ml (Fig. 4d). The two most potent FD varieties for NF-κB p50 deactivation in the nuclear lysate were FDD (10.0%) followed by FDT (6.4%) whilst for NF-κB p65 deactivation they were FDT (39.4%) and FDK (34.9%). The most potent FD varieties for NF-κB p50 downregulation in the cytoplasmic lysate were FDD (12.6%), followed by both FDT and FDK (10.3%), whilst for NF-κB p65 it was FDD (19.5%), and followed by FDK (16.8%) (Table 1).

Compared to LPS control, the co-incubation of LPS and all FD varieties at 5 to 40 μg/ml down-regulated NF-κB p50 gene expression significantly (*p* < 0.001), but this is in contrast to the gene expression of NF-κB p65 i.e. FD varieties at all concentrations did not significantly suppress NF-κB p65 mRNA expression, except FDD at the highest concentration (*p* < 0.001). However, almost all gene expression of NF-κB p65 was reduced although not significant. The two most potent FD varieties for NF-κB p50 and NF-κB p65 gene expression down-regulation were FDK (87.6%), followed by FDT (85.3%) and FDD (1046.1%), followed by FDK (330.8%), respectively **(**Fig. 4e and f, Table 1).

### Effects of FD varieties on eNOS protein and gene expressions

Co-incubation of LPS and FDT at 10 μg/ml (*p* < 0.05) and FDK at 20 μg/ml (*p* < 0.01) enhanced eNOS protein expression. Instead of up-regulating the level of eNOS, FDD and FDI in almost all concentrations reduced the secretion of eNOS. The most potent FD varieties for high expression of eNOS were FDT (15.7%) and FDK (15.1%) **(**Fig. 4g, Table 1).

Co-incubation of LPS and all FD varieties significantly increased the mRNA level of eNOS compared to LPS alone at all different concentrations (*p* < 0.001). The most potent FD varieties for eNOS gene expression up-regulation were FDD (− 99.1%), and FDK (− 100.0%) (Fig. 4h, Table 1).

### Effects of FD varieties on monocytes and LPS-stimulated HCAEC interaction

**Fig. 5:**
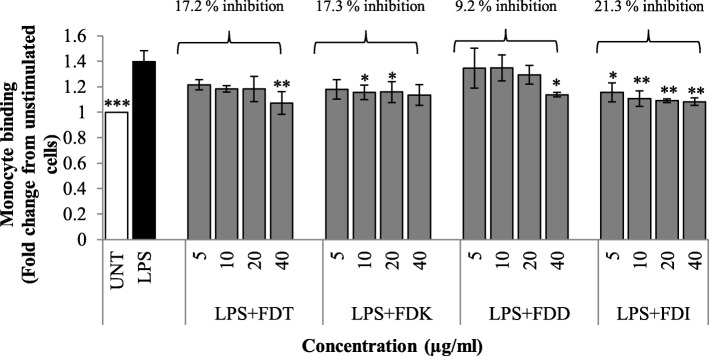
Effects of FD extracts (5–40 μg/ml) on the monocyte-endothelial cell binding assay. Data expressed as mean ± SD. Statistical analysis: ANOVA, post-hoc with Bonferroni correction; *p < 0.05, **p < 0.01 and ***p < 0.001 compared to HCAEC incubated with LPS alone. Abbreviation: UNT (untreated), LPS (lipopolysaccharide), FDT (FD var. *trengganuensis*), FDK (FD var. *kunstleri*), FDD (FD var. *deltoidea*), FDI (FD var. *intermedia*)

Monocyte adhesion assay was performed to explore the effects of FD varieties on monocytes and endothelial cell interactions (Fig. 5). Monocytic U937 cell line showed minimal adherence to the unstimulated HCAEC. After treatment with LPS for 16 h, the adhesion of U937 monocytes to HCAEC was increased markedly. It was found that all FD varieties across all concentrations lead to the reduction of monocyte adhesion to LPS-stimulated HCAEC compared to LPS controls. For FDT and FDD, a significant reduction was only observed at 40 μg/ml (*p* < 0.05 and *p* < 0.01) compared to LPS controls. Co-incubation with 10 and 20 μg/ml of FDK significantly reduced the adhesion of monocytes to the LPS-stimulated HCAEC (*p* < 0.05). Meanwhile, all concentrations of FDI could significantly reduce the number of monocytes adhering to LPS-stimulated HCAEC (*p* < 0.05 and *p* < 0.01). AUC analysis showed that FDI exhibited the highest percentage inhibition of monocytes adhesion (21.3%), followed by FDK (17.3%), FDT (17.2%) and FDD (9.2%).

### Effects of FD extracts of different varieties on the ROS production in LPS and IFN-γ -stimulated RAW 264.7

**Fig. 6:**
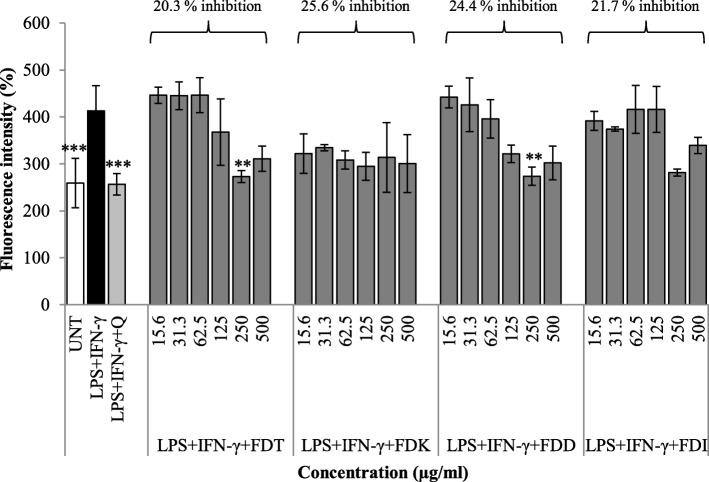
Effects of different concentrations of FD varieties (15.625–500 μg/ml) on the percentage increase of DCF fluorescence in RAW 264.7 cells stimulated with LPS and IFN-γ. Results are expressed as a percentage (%) of fluorescence intensity. Data are expressed as mean ± SD (*n* = 3). Statistical analysis: ANOVA, post-hoc with Bonferroni correction; **p < 0.01 and ***p < 0.001 compared to HCAEC incubated with LPS alone. Abbreviation: UNT (untreated), LPS (lipopolysaccharide), IFN-γ (interferon gamma), Q (Quercetin), FDT (FD var. *trengganuensis*), FDK (FD var. *kunstleri*), FDD (FD var. *deltoidea*), FDI (FD var. *intermedia*)

A high percentage decrease of DCF fluorescence indicates low production of ROS in the cells. Technically, DCFH-DA penetrates cellular membranes and hydrolyses inside the macrophage, causing it to become DCFH or non-fluorescent. DCFH is then fluoresced (known as DCF fluorescence) after being oxidised by ROS. Stimulated RAW 264.7 cells with quercetin (20 μg/ml) were used as a positive control, whilst stimulated cells with medium only (instead of FD varieties) was used as a negative control. Untreated control was HCAEC cell line not stimulated by LPS + IFN-γ and without any treatment. The percentage increase of DCF fluorescence production after treating the cells with 250 μg/ml of FDT and FDD (*p* < 0.01) was significantly lower than the LPS + IFN-γ only group. FDK is the most potent variety in decreasing the production of ROS as evidenced by AUC of 25.6%. The second most potent variety was FDD, with 24.4% inhibition (Fig. 6).

## Discussions

At present, studies on anti-inflammatory effects of FD mainly focused on diabetic prevention and treatment. To the best of our knowledge, this is the first report to describe the anti-atherogenic effect of FD in vitro*.* Although other some have published the anti-inflammatory [17] and anti-oxidant effects of FD [13], its effect against atherogenesis has not been widely studied.

This present study demonstrated that FD extracts of all four species up to 40 μg/ml did not exhibit any effects on the viability of HCAEC. Previous work performed by Abrahim et al. found that FD varieties (small, FDS; medium, FDM) were relatively non-toxic as no IC_50_ was detected up to 72 h incubation in normal liver cell, WRL68 [18]. However, the result of MTT assay from our experiment cannot be compared to the result from the suggested article due to different cell lines and FD varieties being used. This is the first time MTT assay was conducted to examine cytotoxic activity of FD against HCAEC. In general, it was found that all FD varieties were beneficial in inhibiting protein and gene expression of VCAM-1, ICAM-1, E-selectin, IL-6, and NF-κB p65 in LPS-stimulated endothelial cells whilst increasing eNOS secretion. Amongst the four FD varieties, FDK, followed by FDT*,* were the two most potent and effective anti-inflammatory and anti-endothelial activation agents; and the effects were mediated by the NF-κB and eNOS pathways.

High expression levels of cell adhesion molecules (CAMs) such as VCAM-1, ICAM-1, and E-selectin by vascular endothelium are well correlated with atherosclerosis [19, 20]. In atherogenic conditions, CAMs are crucial for leukocyte rolling and arrest, which enhance fatty streak formation [6] while in resting endothelium, E-selectin is scarce. E-selectin is involved in tethering and rolling, which are the early steps of leukocyte recruitment at the endothelial surface [21]. This process creates weak bonds and the adhesion of leukocytes to activated endothelium is further mediated by ICAM-1 and VCAM-1. ICAM-1 induces firm arrest of inflammatory cells at the vascular surface by establishing strong bonds with integrins, while VCAM-1 is favouring firm adhesion by participating in the recruitment of blood cells by activated endothelium [22]. This is why it is important to find a therapy against atherosclerosis that may block adhesion molecules production to disrupt the process of atherogenesis. The co-treatment of all FD varieties of different doses and LPS has different inhibition effects in the production of IL-6, ICAM-1, VCAM-1, and E-selectin. Although other groups have previously published that FD at higher concentrations (50 to 100 μg/ml) led to a reduction in IL-6 protein expression [23]. Zhang et al. reported that vitexin, the main flavonoid found in FD improves oxidised low-density lipoprotein (ox-LDL)-induced endothelial dysfunction by inducing autophagy via AMPK signalling [24]. All FD varieties significantly downregulated ICAM-1 protein expression in almost all concentrations, however, its inhibition in terms of gene expression at certain concentrations was not significant. Unlike other biomarkers, the gene expression level of ICAM-1 did not reflect its protein expression and this variation might be due to their differences in regulatory processes that involve the rates of translation and protein degradation [25, 26]. In addition, ICAM-1 [27] is present in healthy arteries, unlike VCAM-1 [28, 29].

Inflammation and endothelial activation are pivotal in the early development of atherosclerosis. The induction of pro-inflammatory cytokines and adhesion molecules by endothelial cells are mediated by NF-κB activation [30]. The activity of NF-κB is regulated at multiple levels due to its ability to influence numerous gene expressions. Generally, NF-κB p50 and p65 are inactive in the cytoplasm when bound to an inhibitor protein called IκB. However, when the cell is stimulated by LPS, the enzyme IκB kinase (IKK) is activated, causing IκB to be phosphorylated and NF-κB p50 and p65 are then released and translocated into the nucleus [31]. This is where it regulates specific gene expression and lead to physiological responses. In this study, NF-κB p65 protein expression was suppressed with co-incubation of FDT and FDK in almost all concentrations in both nuclear and cytoplasmic lysates. Previous study by Dias et al, reported that another extract, *Euterpe oleracea Martius* polyphenolic enriched with vitexin down-regulated the NF-κB transcription factor and adhesion molecules VCAM-1 and ICAM-1 [32]. Treatment with FD blocked IκB degradation, resulting in high IκB concentration, less NF-κB activation, low NF-κB activity and less gene expression. This also suggests that the reduction of NF-κB in this experiment was likely due to the flavonoid in FD. In addition, Yu et al. [33] reported that the suppression of nuclear translocation of NF-κB subunit p65 and p50 by a phenolic compound named ellagic acid leads to the suppression of VCAM-1 and E-selectin expression, which results in decreased monocyte adhesion. However, this is in contrast with the level of NF-κB p50 protein expression in the nuclear and cytoplasmic lysates, in which all FD varieties showed neutral effects across different concentrations. In terms of gene expression, NF-κB p50 mRNA was significantly down-regulated by all FD varieties but this is not so for NF-κB p65 gene expression. In the case of p65, there was no significant change in the gene expression (except FDD at 40 μg/ml) but reduced expression of the protein could be seen. In contrast, a study by Schwanhausser et al. reported that mRNA levels and translation rates both positively correlate with protein product [34]. We postulate that the effect is not acting at the transcription level, but rather at the translation level, thus, inhibiting the activation of the protein rather than affecting the transcription of the protein. This may be also due to gene expression being suppressed at maximum few hours post-treatment and returned to basal levels before the 16-h incubation end. Further investigation of the possibility of different NF-κB subunits regulation is required. So far, p65 received the most attention in studies of NF-κB phosphorylation, whereas phosphorylation of p50 is much less well understood [35].

Recruitment of monocytes is also an important event in the process of atherosclerotic plaque formation. This process is influenced by chemoattractants, adhesion molecules and some receptors. Monocytes differentiate into macrophages in the vessel wall and start to take up lipids, which result in the transformation of macrophage into foam cells [28]. Scarce data have been reported on the effects of FD varieties on monocyte adhesion. Previously, a study by Chen et al. showed that pre-treated HCAEC with other compounds, namely eicosapentaenoic acid (EPA) or docosahexaenoic acid (DHA) could reduce monocyte adhesion in response to ox-LDL-stimulated HCAEC [36]. This finding is in parallel with the present study, where the untreated group showed minimal binding to U937 cells, whereas treatment with LPS markedly increased the adhesion of monocytes to HCAEC. This study also revealed that all four FD varieties could inhibit monocytes adhesion to endothelial cells, with FDI followed by FDK exhibiting the highest percentage inhibition of monocytes adhesion compared to the other varieties. The increased potency of FD varieties is likely to be due to greater beneficial effects in terms of inhibition of adhesion molecules.

The presence of anti-oxidants in natural products is not only good for health but is also believed to serve therapeutic benefits particularly in human atherosclerotic cardiovascular disease, as oxidative stress is closely related to inflammation and endothelial dysfunction [37]. Therefore, the inhibition of the activation process may be useful as a potential therapeutic target [38]. NO is important in regulating vascular homeostasis. When endothelial cells are under oxidative stress there will be less NO production caused by eNOS uncoupling, and increase excessive production of ROS [39]. This enzymatic uncoupling is often accompanied by tetrahydrobiopterin (BH4) deficiency [40], in which BH4 is an important cofactor for NO synthesis. The dysfunction of eNOS is linked to the changes in the expression of VCAM-1, ICAM-1 and E-selectin [41–43], which will then promote the adherence of leukocytes to the endothelium. In this study, eNOS protein expression level was measured and it was found that all varieties except FDD raised the expression of eNOS. However, only FDT at 10 μg/ml and FDK at 20 μg/ml remarkably elevated the expression of eNOS when compared to LPS-stimulated controls. With regards to gene expression of eNOS, the mRNA levels increased significantly from LPS-stimulated controls when supplemented by all varieties of FD. Previous in vivo study using bioactive compound oligomeric proanthocyanidins from *Rhodiola rosea* by Zhou et al. also reported that there was increased expression of eNOS in atherosclerosis rats in the treatment group compared to the placebo group [44]. Similar finding reported by Muid et al. in which eNOS production was significantly increased by the co-incubation of LPS and tocotrienol (TCT) isomers: γ-TCT (5 and 10 μM), and δ-TCT (1.3 and 2.5 μM) in human umbilical vein endothelial cells (HUVEC) compared to LPS controls [45]. The formation of intracellular ROS in the macrophage was determined by DCFH-DA assay. In this study, DCF fluorescence was significantly inhibited when the stimulated cells were incubated with 250 μg/ml of FDT and FDD; FDK and FDD were identified as the two most potent varieties in lowering the ROS production. This finding is important since increased numbers of macrophages populating the atherosclerotic plaque will also increase ROS production, thus leading to the accumulation of oxidised LDL. Hence, this finding reflects the effectiveness of FD in preventing endothelial dysfunction through suppressing oxidative stress via eNOS pathway; the presence of FD may lower the production of ROS, as well as oxidised LDL. This also suggests that the FD varieties at appropriate doses are effective in preventing and inhibiting oxidative damage to lipids and protein. The potent anti-oxidant activity of FD variants reported by the present study is in agreement with a study by Misbah et al., although different anti-oxidant assays were used. The author reported that aqueous extracts of two fruit varieties of FD (var. *angustifolia* and var. *kunstleri*) have shown anti-diabetic and anti-oxidant activities in in vitro studies, with anti-oxidant activities that might be asserted by phenolic content evident by the positive correlation of anti-oxidant activity and the crude extracts [46].

There are many established therapies in the treatment of atherosclerotic cardiovascular disease such as statins, ezetimibe, β-blockers, aspirin, angiotensin receptor blockers and very recently proprotein convertase subtilisin/kexin type 9 (PCSK9) inhibitor. Despite all the available therapies, most of them focus on inhibiting hypertension and hyperlipidemia or controlling hemostasis. Only a few treatments are targetting the inflammatory mechanisms that are essential in the progression of the disease [47]. There are also multiple cases reported, where patients that consumed established treatment such as a statin developed side effects and unable to achieve the target LDL-c levels. Kaliora and Dedoussis remark that it is necessary to find other treatment alternatives because lipid-lowering such as statins are not only effective in the treatment of CVD but also can cause long-term side effects [48]; established treatments for treating CHD are not optimal. The use of FD can be utilised as an adjunct treatment to standard therapy in CHD patients, as well as patients with drugs or statin intolerant. Although the numbers of deaths have declined for decades, CHD still remains the number one killer worldwide and in Malaysia. In addition, medication costs for atherosclerosis-related cardiovascular disease is definitely a burden to the nation’s economy; FD as a cheaper option.

## Conclusion

All FD varieties suppressed endothelial activation, inflammation, monocyte binding activity and oxidative stress in stimulated HCAEC; with FDK*,* followed by FDT exhibiting the most potent effects. The anti-atherogenic actions were mediated via the NF-κB and eNOS pathways. These findings suggest that FD has anti-atherogenic properties and is a starting point towards determining its potential use in humans as a novel, natural atheroprotective agent. Future studies are warranted to explore the in vivo effects of the best FD variety in the prevention of atherogenesis.

## Data Availability

Not applicable

## Abbreviations

ANOVA: Analysis of variance
ATCC: American Type Culture Collection
AUC: Area under the curve
AuRIns: Atta-ur-Rahman Institute of Natural Product Discovery
BH4: Tetrahydrobiopterin
CAM: Cell adhesion molecule
CO_2_: Carbon dioxide
DCFH-DA: 2′-7′-dichlorofluorescein diacetate
DHA: Docosahexaenoic acid
DMEM: Dulbecco’s Modified Eagle Media
DMSO: Dimethyl sulfoxide
EGM: Endothelial growth medium
eNOS: Endothelial nitric oxide synthase
EPA: Eicosapentaenoic acid
E-selectin: Endothelial-leukocyte adhesion molecule-1
FBS: Fetal bovine serum
FD: *Ficus deltoidea*
FDD: FD var. *deltoidea*
FDI: FD var. *intermedia*
FDK: FD var. *kunstleri*
FDT: FD var. *trengganuensis*
GAPDH: Glyceraldehyde-3-phosphate dehydrogenase
GUSB: Glucuronidase beta
HCAEC: Human coronary artery endothelial cell
HPRT1: Hypoxanthine phosphoribosyltransferase 1
HUVEC: Human umbilical vein endothelial cell
ICAM-1: Intercellular cell adhesion molecule-1
IFN- γ: Interferon gamma
IKK: IκB kinase
IL-6: Interleukin-6
LPS: Lipopolysaccharide
MTT: 3-(4,5-dimethylthiazol-2-yl)-2,5-diphenyl tetrazolium bromide
NF-kB: Nuclear factor-κB
NO: Nitric oxide
ox-LDL: Oxidised low-density lipoprotein
PBS: Phosphate-buffered saline
PCSK9: Proprotein convertase subtilisin/kexin type 9
ROS: Reactive oxygen species
SAPE: Streptavidin-conjugated R-Phycoerythrin
SD: Standard deviation
UiTM: Universiti Teknologi MARA
UniSZA: Universiti Sultan Zainal Abidin
VCAM-1: Vascular cell adhesion molecule-1
